# A *PITX2* splice-site mutation in a family with Axenfeld-Rieger syndrome leads to decreased expression of nuclear PITX2 protein

**DOI:** 10.1007/s10792-021-01704-5

**Published:** 2021-01-25

**Authors:** Feng Zhang, Lusi Zhang, Li He, Mengdan Cao, Yuting Yang, Xuanchu Duan, Jingming Shi, Ke Liu

**Affiliations:** 1 Department of Ophthalmology, Second Xiangya Hospital, Central South University, Changsha, 410011 Hunan China; 2 Department of Ophthalmology, Third Xiangya Hospital, Central South University, Changsha, Hunan China; 3 Department of Medicine, University of Alabama At Birmingham, Birmingham, AL USA; 4 Aier School of Ophthalmology, Central South University, Changsha, Hunan China; 5 Changsha Aier Eye Hospital, Changsha, Hunan China

**Keywords:** Axenfeld-Rieger syndrome, *PITX2*, Splice-site variation, Heterogeneity, Glaucoma

## Abstract

**Purpose:**

Axenfeld-Rieger syndrome (ARS) is an autosomal dominant disorder characterized by ocular anterior segment abnormalities. In the current study, we describe clinical and genetic findings in a Chinese ARS pedigree.

**Methods:**

An ARS pedigree was recruited and patients were given comprehensive ophthalmic examinations and general physical examinations. DNA from the proband II:2 was used for exome sequencing. Sanger sequencing was utilized to identify and validate *PITX2* variations. qPCR and western blotting were performed to detect *PITX2* expression in immortalized peripheral blood lymphocytes.

**Results:**

All affected family members showed typical ocular abnormalities, including iris atrophy, corectopia, shallow anterior chamber, complete or partial angle closure, and advanced glaucoma. They also exhibited systemic anomalies, such as microdontia, hypodontia, and redundant periumbilical skin. A heterozygous splice-site variation c.390 + 1G > A in *PITX2*, which might lead to a truncated PITX2 protein (p.Val131IlefsX127), was found in the proband. Sanger sequencing validated that the variation completely co-segregated with the ARS phenotype within this family and was absent in 100 unrelated controls. Western blotting revealed that the nuclear PITX2 protein was significantly decreased in patients compared with controls. Nonetheless, there was no significant difference in the total PITX2 protein level, consistent with qPCR results showing no alteration in *PITX2* mRNA levels in the patient group.

**Conclusions:**

*PITX2* c.390 + 1G > A (p.Val131IlefsX127) was a novel genetic etiology of the ARS pedigree. The mutation leads to decreased nuclear *PITX2*, indicating lower transcriptional activity.

## Introduction

Axenfeld-Rieger syndrome (ARS) is an autosomal dominant hereditary disease with highly genetic heterogeneity [1]. The major candidate genes are paired-like homeodomain 2 (*PITX2*), located at 4q25, which plays a fundamental role in genetic control of ocular anterior segment development [2], and forkhead box C1 (*FOXC1*), located at 6p25, which acts as an important regulator of cell migration and differentiation during embryogenesis [3]. Mutations in these two genes are found in 40%–70% of ARS patients [4, 5]. *PITX2* mutations are commonly associated with ocular, dental, and umbilical anomalies, whereas *FOXC1* mutations are mostly associated with isolated ocular or ocular, heart, and/or hearing defects [6]. Other genetic causes, such as a mutation at locus 13q14 [7] might also be associated with ARS.

Clinical phenotypes of the eye in ARS patients are heterogeneous, which can present as posterior embryotoxon, iridogoniodysgenesis, polycoria, corectopia, or iris stromal hypoplasia. Glaucoma, secondary to maldevelopment of aqueous humor drainage structures, is the most serious consequence of ARS; approximately 50% of ARS patients progress to glaucoma [8]. Onset of secondary glaucoma typically occurs before the teenage years [9].

The current study describes the clinical phenotypes and genetic characterization of a Chinese family with ARS who harbor a *PITX2* c.390 + 1G > A (p.Val131IlefsX127) mutation. Western blotting and quantitative real-time PCR (qPCR) indicated decreased levels of nuclear PITX2 protein in peripheral blood lymphocytes (PBL), but total PITX2 levels were unchanged.

## Material and methods

### Subjects

A Chinese Han pedigree with four affected individuals was recruited and peripheral blood samples were collected. The study adhered to the principles of the declaration of Helsinki; clinical protocols were approved by the Second Xiangya Hospital Institutional Review Board. Written informed consent was obtained from all included subjects.

Patients underwent basic ophthalmic examinations that comprised evaluation of best-corrected visual acuity (BCVA), Goldman applanation tonometry, slit-lamp biomicroscopic examination, and fundus examination. Other necessary examinations were also performed, including fundoscopy (non-mydriatic retinal camera, TRC-NW300; Topcan, Itabashi-ku, Tokyo, Japan), anterior segment photography (SL-D4; Topcan, Itabashi-ku), ultrasound biomicroscopy (UBM), and assessment of visual field defects (Humphrey Visual Field Analyzer; Carl Zeiss Humphrey Systems, Dublin, CA, USA).

## Targeted exome sequencing

Genomic DNA was extracted according to the standard phenol–chloroform method. DNA of the proband II-2 was used for 90-cycles of paired-end exome sequencing performed on Illumina HiSeq2500 Analyzers (Illumina, San Diego, California, USA). Base-calling and calculation of quality values for every base were performed by pipeline software version 1.3.4 (Illumina). Reads were aligned to human reference genome GRCh37 using the Burrows-Wheeler Aligner (BWA; http://bio-bwa.sourceforge.net/) [10]. Single nucleotide variants (SNVs) and insertions and deletions (indels) were identified with SOAPsnp (http://soap.genomics.org.cn/soapsnp.html) and samtools (http://samtools.sourceforge.net/). SNVs and indels with read depth ≥ 8 × and quality ≥ 30 were reserved for subsequent analyses. Polymorphic SNVs were excluded based on the dbSNP database and the 1000 genomes annotation. SNVs and indels affecting coding sequences were annotated with Annotate Variation (ANNOVAR, http://annovar.openbioinformatics.org).

## Sanger sequencing of implicated gene and splice site prediction

Sanger sequencing of the PCR amplicons was used to validate the pathogenic *PITX2* (NM_001204397) mutation identified via exome sequencing. Sequences of the primers used for PCR were: forward primer (F), 5′-GACGGGAAAGTGTGTGTGTTT-3′, and reverse primer (R), 5′-GAGGGAACTGTAATCTCGCAAC-3′. PCR was performed in a 10 µl reaction mixture using 2 × Takara Taq™ HS Perfect Mix (Takara Biotechnology, Dalian, China). Amplification conditions consisted of an initial denaturation at 94 °C for 30 s, followed by 33 cycles of denaturation at 94 °C for 5 s, annealing at 60 °C for 20 s, and extension at 72 °C for 20 s. Final extension was performed at 72 °C for 7 min. NetGene2 (http://www.cbs.dtu.dk/services/NetGene2/) was used to predict the donor and acceptor splice sites of the mutant *PITX2* sequence.

## Construction of immortal peripheral blood lymphocytes (PBL)

The mixture, which contained 2 mL heparin sodium treated blood, 2 mL RPMI-1640 basic medium and Ficoll-Paque PLUS (GE Healthcare, Chicago, IL, USA), were centrifuged at 2500 rpm for 10 min to isolate lymphocytes from human peripheral blood. The lymphocytes layer was carefully extracted with a Pasteur pipet and rinsed with RPMI-1640 basic medium three times. Following 1000 rpm centrifugation for 5 min, the pellet containing lymphocytes was resuspended in 1.5 mL lymphocyte culture medium containing 75% RPMI-1640 and 25% FBS (Gibco, NY, USA) and cultured in a T25 flask at 37 °C in a 5% CO_2_ atmosphere. B95-8 cells were cultured in a T25 flask for 5–7 days without changing the medium. Cells and medium were collected in a 15 ml conical tube and cryopreserved in −80 °C. When using EB virus, the cellular mixtures were frozen and thawed repeatedly three times, followed by centrifugation at 3000 rpm for 3 min. The supernatant was then collected for PBL infection by adding 200 µg/ml CyA (0.1 ml; Sigma-Aldrich, St. Louis, MO, USA) and 1.5 ml B95-8 cell derived EB virus into the primary PBL. Lymphocyte culture medium was added to cells depending on their growth until the medium volume reached 20 ml, after which half of the medium was replaced every 2–4 days. After culturing for 3–4 weeks, an immortal PBL line was obtained.

## RNA extraction and qPCR measurement

Total RNA was extracted from immortal lymphocytes derived from two patients and three controls using Trizol reagent (Life Technologies, NY, USA). cDNA was synthesized from 1 μg total RNA using the RevertAid First Strand cDNA Synthesis Kit (Thermo Scientific, Waltham, MA, USA). qPCR was performed on an Applied Biosystems® StepOne™ Plus Real-Time PCR System (Thermo Scientific, Waltham, MA, USA) with Maxima SYBR Green qPCR Master Mixes (Thermo Scientific). Relative expression of *PITX2* was normalized to β-actin and analyzed by the comparative Ct method. Primers used for qPCR were: *PITX2* (after the mutant site): forward primer, 5′-TACCTGTCCCTGTCACTCTTGA-3′ and reverse primer, 5′-AAGAACCCCTCCAATAAGGAAA-3′, β-actin, forward primer, 5′-CACGATGGAGGGGCCGGACTCATC-3′ and reverse primer, 5′-TAAAGACCTCTATGCCAACACAGT-3′. A two-tailed Student’s t test was used to determine the significance of differences between the two groups. Data is presented as mean ± SD.

## Western Blotting and nuclear extraction

Nuclear extraction from PBL was performed using the NE-PER™ Nuclear and Cytoplasmic Extraction Reagents (Thermo Scientific). Cell lysates and PBL nuclei were prepared with SDS sample buffer (63 mM Tris–HCl, 10% Glycerol, 2% SDS, 0.0025% Bromophenol Blue, pH 6.8) supplemented with 1% protease inhibitor cocktails (Sigma-Aldrich, St. Louis, MO, USA). After ultra-sonication, the lysate was centrifuged at 13,000 rpm for 10 min at 4 °C to collect the supernatant. The protein concentration was measured using the Pierce BCA™ protein assay kit (Thermo Scientific). Total cellular (20 ug) and nuclear (40 ug) protein were loaded onto SDS-PAGE gels. Proteins were transferred to a PVDF membrane, blocked with 5% non-fat milk in 0.1% PBST (0.1% Triton-X 100 in 1 × PBS), and blotted with primary antibodies (anti-PITX2 antibody, Abcam, Cambridge, UK; anti-PCNA antibody, Boster Biotechnology, China; anti-GAPDH antibody, Sigma-Aldrich) overnight at 4 °C. After washing three times in 0.1% PBST for 30 min, membranes were incubated with secondary HRP-conjugated antibodies (Kangwei biotech, China) for 1 h at room temperature. After washing with 0.1% PBST three times for 10 min each, membranes were treated with HRP substrates and visualized on a FluorChem FC3 system (ProteinSimple, California, USA).

## Results

### Clinical findings

**Fig. 1:**
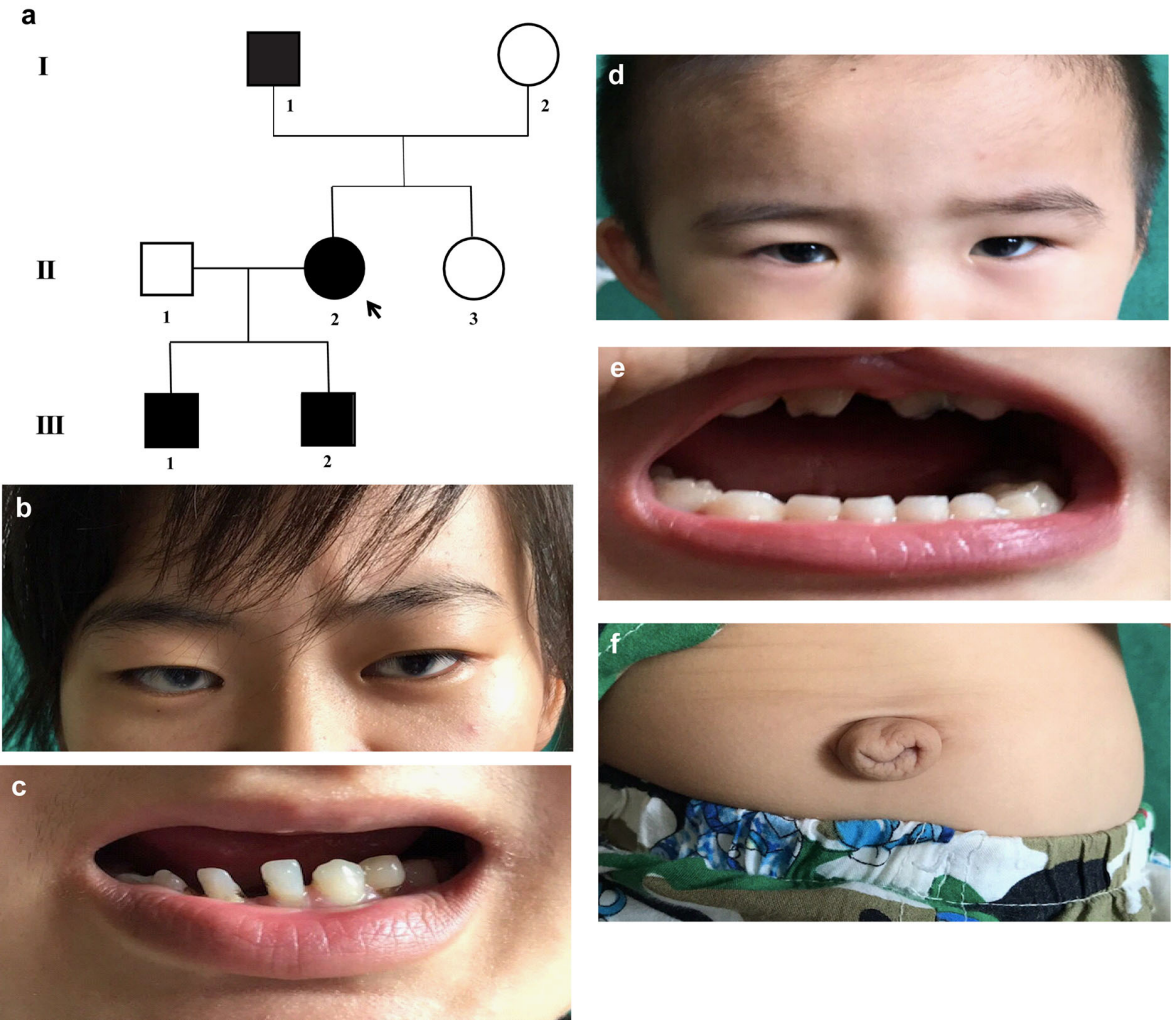
General phenotypes of the proband. **a** The AR pedigree. Roman numerals refer to generations and individuals within a generation were numbered from left to right. Proband is noted with an arrow. Filled symbols refer to ARS patients, open symbols refer to unaffected individuals. **b** The ocular features of the proband. **c** Intraoral view of the proband. **d** The ocular features of the patient III:1. **e** Intraoral view of patient III:1. **f** Protuberant umbilicus in patient III:1

**Fig. 2:**
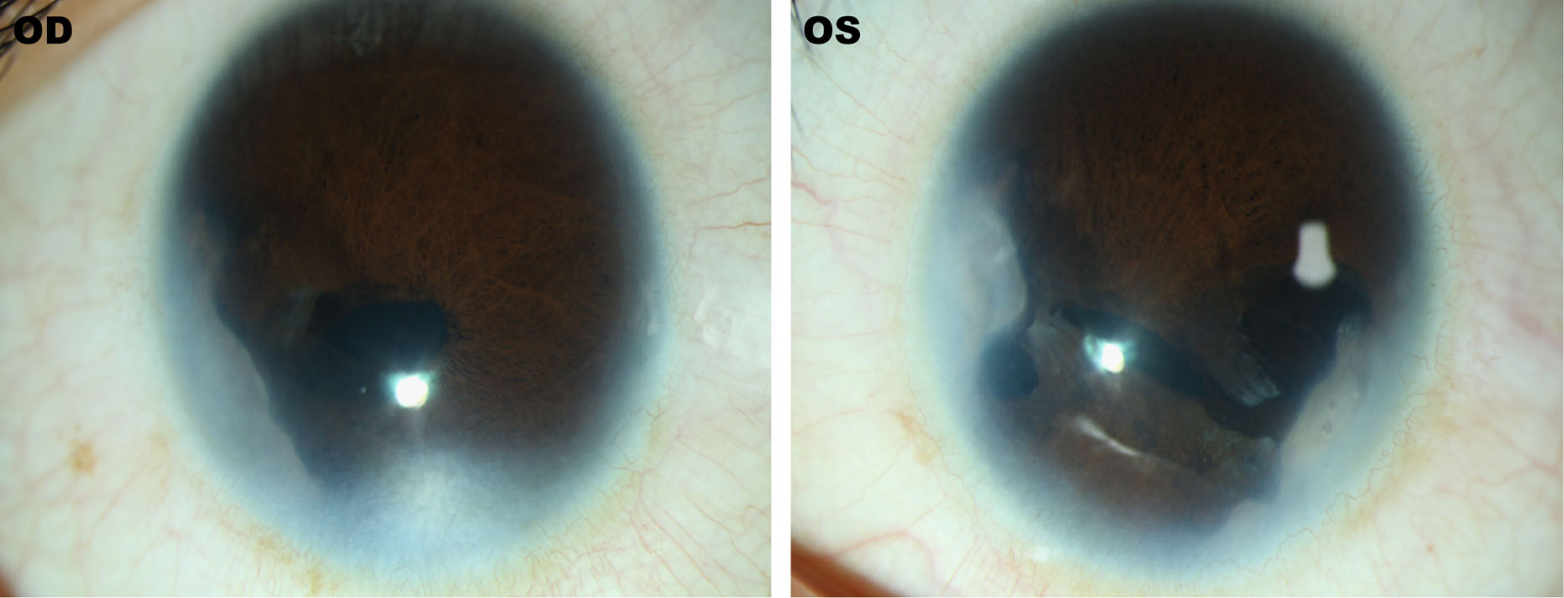
Clinical ocular symptoms of the proband. The bilateral eyes have small corneas, irises atrophy, corectopia, polycoria, and synechia. OD, right eye; OS, left eye

We described a three-generation pedigree (Fig. 1a) in which four of seven individuals were diagnosed with Axenfeld-Rieger syndrome (ARS). The proband II:2 in our study is a 26-year-old female who presented with gradual dimness of vision over five years. Her best-corrected visual acuity was 0.2 in the right eye and 0.1 in the left eye. Her intraocular pressure (IOP) was 29 mmHg eye and 25 mmHg in the right and left eye, respectively. She presented with small cornea, iris atrophy, corectopia, polycoria, and synechia in both eyes (Fig. 1b, Fig. 2). Moreover, she had typical craniofacial features of ARS, such as prominent forehead, telecanthus, and a flattened mid-face with a broad, flat nasal bridge (Fig. 1b), thin upper lip, hypodontia and microdontia (Fig. 1c). Her elder son (III:1) who is five years old was diagnosed with secondary glaucoma at age three. His intraocular pressure was 13 mmHg and 12 mmHg in the right and left eye, respectively, and he was being treated with 0.5% Timolol twice a day. He also had clinical features typical of ophthalmic and craniofacial disorders (Fig. 1d, e) as well as redundant peri-umbilical skin in his abdomen (Fig. 1f). The proband’s father (I:1), who is now 52 years old, had had no light perception for 10 years and was diagnosed with secondary glaucoma at age 36. His IOP was 31 mmHg and 32 mmHg in the right and left eye, respectively, and he was being treated with three IOP-lowering medicines. He also had clinical features that could be diagnosed as ARS. There were no clinical features in other individuals in the pedigree.

## Exome sequencing identified *PITX2* c.390 + 1G > A (p.Val131IlefsX127) as the genetic basis of ARS

**Fig. 3:**
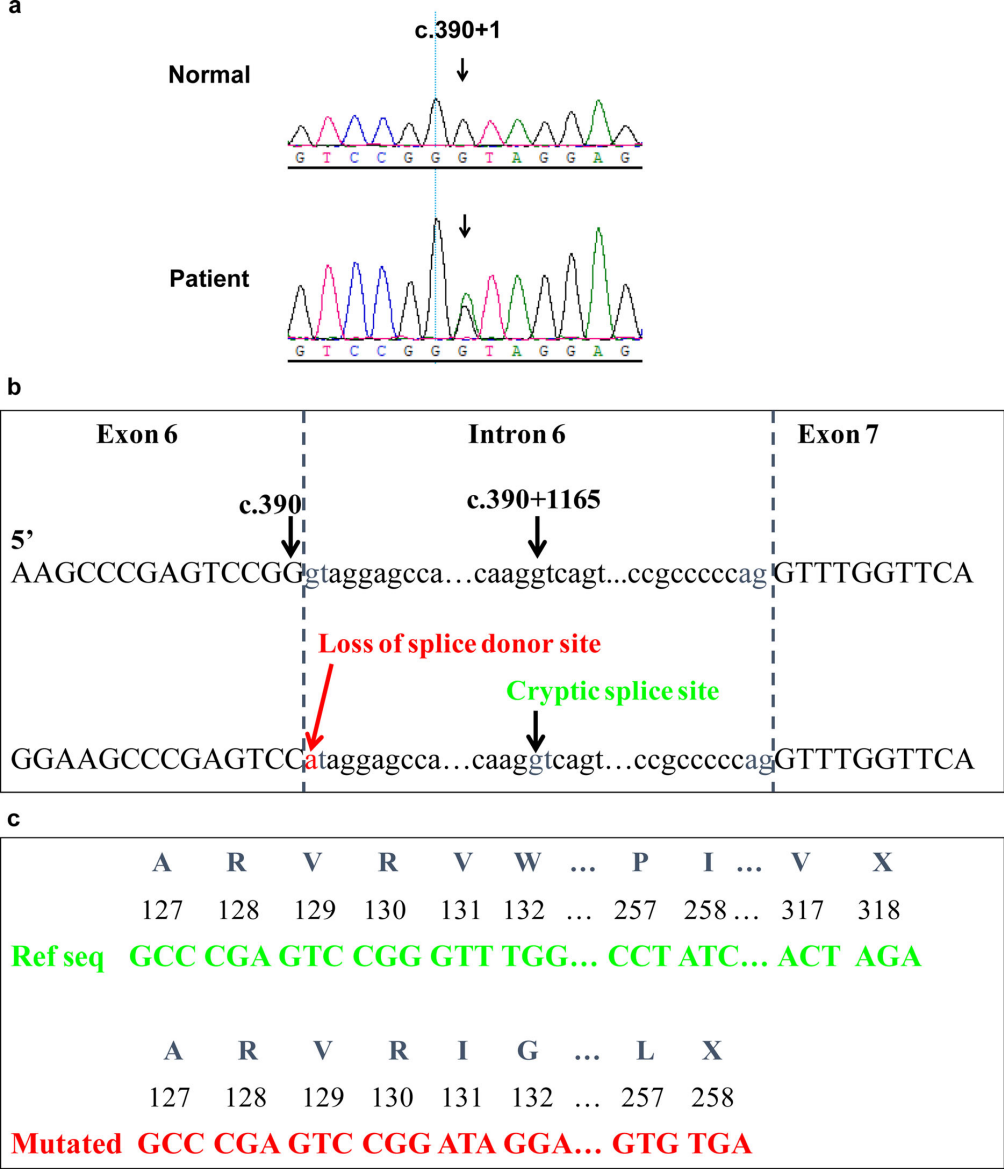
DNA sequence analysis of the *PITX2* c.390 + 1G > A (p.Val131IlefsX127) mutation. **a** Sanger sequencing results of the pedigree. The arrow refers to the mutant base. **b** The splice site change caused by DNA mutation. c.390 + 1G > A ruins the former splice donor site in c.390 and introduces a new splice donor site in c.390 at c.390 + 1165, which brings part of intron 6 into the mutant transcript (Fig 3b). **c** The splice site changes give rise to a shift in the reading frame and introduce a stop codon at position 258

To identify the possible genetic mutation within the pedigree, we chose DNA from the proband II:2 for exome sequencing. A heterozygous c.390 + 1G > A substitution in the splice donor site of *PITX2* intron 6 was detected. All family members were then screened for a mutation at this site via Sanger sequencing. The same was detected in I:1, III:1 and III:2 (Fig. 3a). All but III:2, who showed a normal phenotype, were diagnosed with ARS. Since III:2 is only 3 years old and the disease usually occurs after the teenage years, it is possible that he could exhibit an ARS phenotype as he ages. The c.390 + 1G > A mutation was not detected in any of 100 control individuals from outside the pedigree.

NetGene2 was used to predict splice site changes resulting from the mutation detected in the pedigree. As a result of the mutation, the guanine at c.390 + 1 of PITX2 is replaced by an adenine, causing the insertion of partial intron 6 into the mutant transcript (Fig. 3b), which is predicted to create a shift in the reading frame and introduce a stop codon at position 258 (Fig. 3c). This genetic result suggests that *PITX2* c.390 + 1G > A may be the etiology of the ARS in this family.

## Decreased nuclear PITX2 expression level

**Fig. 4:**
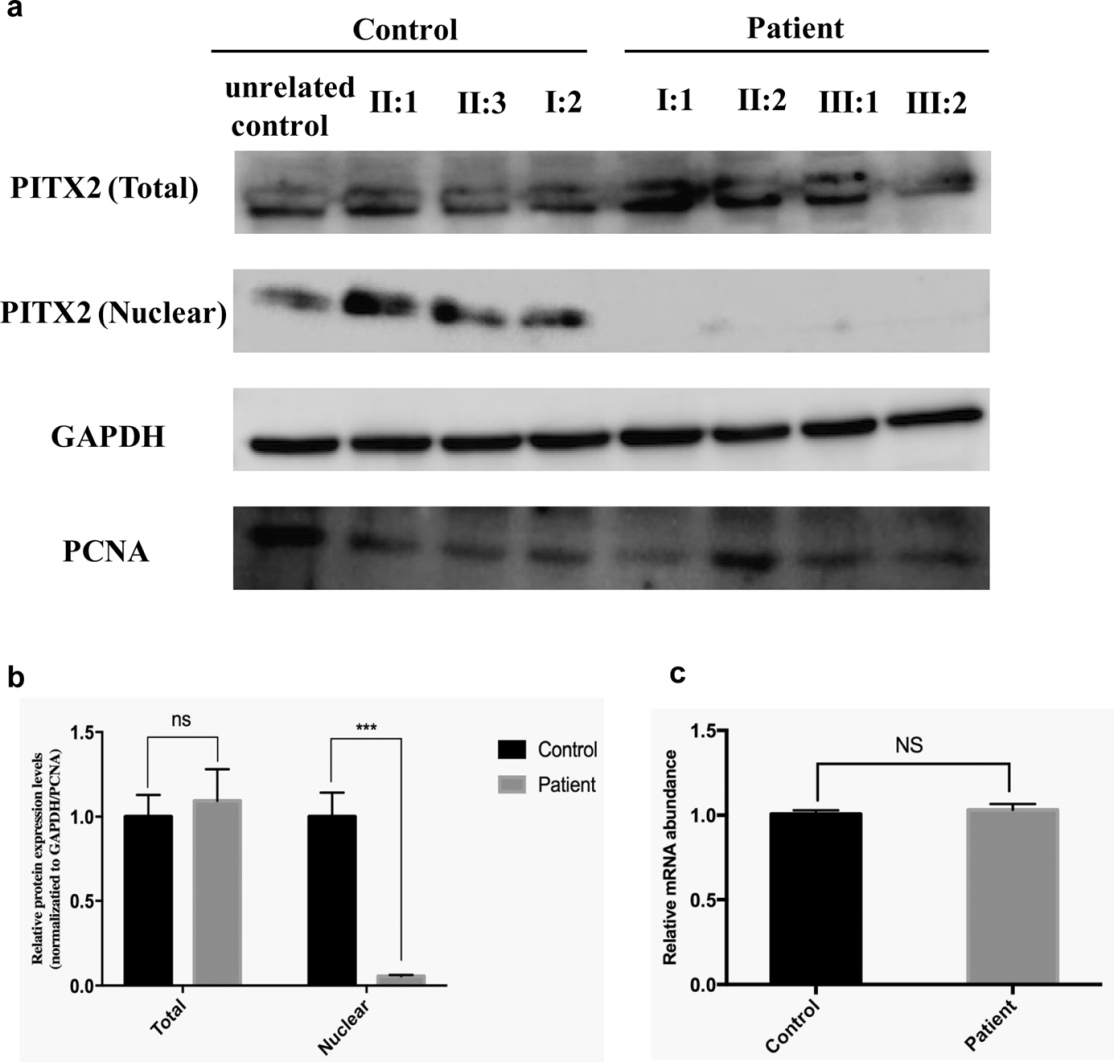
Decreased nuclear PITX2 expression in patients. **a** Immunoblotting shows that total PITX2 levels in patients and controls are not significantly different. **b** The quantitative results of immunoblotting. **c** qPCR result shows that there is no difference between patients and controls in *PITX2* transcription in immortalized peripheral blood lymphocytes. ***, *p* < 0.001; ns, no significant difference

To further explain the effect of the mutant PITX2 protein in the ARS phenotype, we constructed immortal PBL lines from all seven family members (four patients and three normal members) as well as from one unrelated normal control, to assay the expression level and localization of PITX2. As is shown in Fig. 4c, qPCR of PBL mRNA showed no significant difference in *PITX2* transcription between patients and controls (1.075 ± 0.022 and 1.032 ± 0.035, respectively; *p* = 0.2652). Moreover, immunoblotting (Fig. 4a and b) suggested no significant differences among patients and controls in the total cellular PITX2 protein level (1.094 ± 0.187 and 1 ± 0.129, respectively; p = 0.4004), which is accordance with our qPCR results. Considering that PITX2 is a crucial transcriptional factor in ocular development and needs to translocate to the cellular nucleus for normal function, we detected the PITX2 protein level in the nucleus. The PITX2 mutation led to decreased nuclear PITX2 protein levels compared to controls (0.0552 ± 0.00808 and 1.000 ± 0.143, respectively; *p* = 0.0008) (Fig. 4a, 4b). Taken together, the dislocation of PITX2 protein might cause insufficient functional PITX2 protein in nucleus, which in turn caused ocular anterior segment developmental abnormalities.

## Discussion

ARS, which is caused by mutations of either PITX2 or FOXC1, exhibits considerable phenotypic heterogeneity. We now report an ARS pedigree with cosegregating mutations in *PITX2* that was associated with typical anterior segment phenotypes, glaucoma, and craniofacial features. The age of optical symptoms presentation was younger in the second generation (II:2 and III-1) than the first (I:2). The IOP was easily controlled in all patients, indicating that the phenotype resulting from this mutation is relatively mild.

PITX2 is a homeodomain transcription factor that regulates the development of the ocular anterior segment [11], branchial arches [12], heart [13], and pituitary [14]. PITX2 mutations have been associated with a series of anterior segment malformations including ARS, Peter’s-like anomaly, iris hypoplasia/iridogoniodysgenesis syndrome, and ring dermoid of the cornea [5]. It has been reported to be a key effector of anterior segment patterning through retinoic acid and Wnt signaling [15]. In developing murine eyes, the expression pattern of *PITX2* is initiated in the periocular mesenchyme, retained in mesenchyme derived corneal endothelium and TM, and finally restricted to the angle [16, 17]. PITX2 and FOXC1 might form a complex within the nucleus on the chromatin [18]. The *PITX2* c.390 + 1G > A mutation we detected in this study is predicted to produce a truncated protein, which leads to the ARS pathology. However, in the current study, we observed both normal levels both mRNA and protein expression from *PITX2* in patient-derived PBLs. It is probable that the normal copy of the *PITX2* gene can compensate for gene expression. Moreover, the mutant PITX2 present in the pedigree exerted a dominant negative effect over the wild-type allele. While there was no difference in the level of cellular PITX2 in affected and unaffected pedigree members, those with the c.390 + 1G > A mutation had lower levels nuclear PITX2. As the subcellular localization of PITX2 is required for ocular development, the lower levels functional protein in the nucleus may be the cause of ARS phenotype in our pedigree.

In summary, we report a novel heterozygous splice site mutation in *PITX2* in a family with variable ocular phenotypes. A correlation between the *PITX2* mutation and phenotypic severity was observed, and a disturbed PITX2 nuclear localization was identified in affected individuals. Further study is warranted to determine the cause of the impaired PITX2 protein nuclear localization in this patient cohort.
